# The Stress Hyperglycemia Ratio as a Predictor of Clinical Outcomes in Acute Pancreatitis: A Retrospective Cohort Study

**DOI:** 10.3390/jcm14144970

**Published:** 2025-07-14

**Authors:** Ping Zhu, Xinwei Wang, Cheng Hu, Xiaoxin Zhang, Ziqi Lin, Tao Jin, Lan Li, Na Shi, Xinmin Yang, Wei Huang, Qing Xia, Lihui Deng

**Affiliations:** West China Centre of Excellence for Pancreatitis, Institute of Integrated Traditional Chinese and Western Medicine, West China Hospital, Sichuan University, Chengdu 610041, China; zhuping@wchscu.cn (P.Z.); 2023224020203@stu.scu.edu.cn (X.W.); hucheng@wchscu.cn (C.H.); zhangxiaoxin@wchscu.cn (X.Z.); linziqi@wchscu.cn (Z.L.); jintao@wchscu.cn (T.J.); lilan@wchscu.cn (L.L.); nashi@scu.edu.cn (N.S.); yangxinmin@wchscu.cn (X.Y.); dr_wei_huang@scu.edu.cn (W.H.); xiaqing@medmail.com.cn (Q.X.)

**Keywords:** acute pancreatitis, blood glucose, stress hyperglycemia ratio, stress hyperglycemia, clinical outcomes

## Abstract

**Background**: The stress hyperglycemia ratio (SHR) has emerged as a promising biomarker for assessing stress-induced hyperglycemia (SH) but has not been evaluated in patients with acute pancreatitis (AP). This study investigates the role of the SHR in predicting adverse clinical outcomes in patients with AP. **Methods**: Adult patients with AP who were admitted within 72 h of the onset of abdominal pain were screened in the database. Eligible patients with glycated hemoglobin (HbA1c) and blood glucose were analyzed. The SHR was calculated using admission blood glucose and HbA1c levels. Patients were categorized into four groups: SHR1 (≤1.03), SHR2 (1.04–1.25), SHR3 (1.26–1.46), and SHR4 (≥1.47). The primary outcome was persistent organ failure (POF). The secondary outcomes included acute peripancreatic fluid collection (APFC) and high-dependency unit/intensive care unit (HDU/ICU) admission. Restricted cubic spline (RCS) analysis was used to assess nonlinear associations and identify SHR threshold values. Univariable and multivariable logistic regression models were used to adjust for potential confounders and evaluate the relationship between the SHR and clinical outcomes. **Results**: A total of 486 patients with AP were included in this study, comprising 85 with POF and 401 without POF. SHR levels and severity were significantly correlated, with the highest quartile in the greatest proportion of severe acute pancreatitis (SAP). Higher SHR levels were significantly associated with an increased risk of POF, APFC, and HDU/ICU admission. RCS analysis revealed a nonlinear relationship between the SHR and APFC (*p* = 0.009). Based on the RCS and quartile analysis, SHR > 1.25 was identified as the threshold for increased risk. After adjusting for confounders, SHR > 1.25 remained independently associated with higher risks of POF (OR: 2.49, 95% CI: 1.39–4.46, *p* = 0.002), APFC (OR: 2.85, 95% CI: 1.92–4.24, *p* < 0.001), and ICU admission (OR: 1.74, 95% CI: 1.12–2.69, *p* = 0.013). **Conclusions**: The SHR is independently associated with adverse clinical outcomes in AP, including POF, APFC, and HDU/ICU admission. These findings suggest that the SHR may serve as a valuable biomarker for risk stratification and early intervention in AP management.

## 1. Introduction

Acute pancreatitis (AP) is an inflammatory disease of the pancreas [1] with rising incidence worldwide [2,3], imposing considerable clinical and healthcare burdens. Severe acute pancreatitis (SAP), defined by persistent organ failure (POF) [4], is associated with a high mortality rate of 36% to 50% [5]. However, effective early prognostic markers are still lacking to guide timely clinical decisions and improve outcomes.

Stress hyperglycemia (SH), a transient rise in blood glucose during acute illness [6], frequently occurs in AP due to endocrine dysfunction and systemic stress responses [7,8]. Despite evidence [7,9,10,11,12] demonstrating an association between stress hyperglycemia (SH) and adverse clinical outcomes in patients with AP, a consensus on its diagnostic criteria remains lacking.

In recent years, researchers proposed the stress hyperglycemia ratio (SHR) as a new indicator to assess the level of acute hyperglycemia [13]. The SHR is defined as the ratio of admission blood glucose to glycated hemoglobin (HbA1c), where HbA1c is counted as the baseline glucose level to explain and evaluate the effect of background blood glucose. As the SHR integrates both the acute metabolic stress response and the underlying chronic glycemic regulation, it is considered a more stable and reliable indicator than conventional blood glucose levels, which can be affected by short-term fluctuations in insulin sensitivity and acute stress conditions. Nowadays, the SHR has demonstrated robust prognostic value in various diseases, including cardiovascular diseases [14,15,16,17,18,19,20], stroke [21,22,23,24], sepsis [25,26,27], and pneumonia [28]. However, the role of the SHR in AP has not yet been explored.

In this study, we aim to evaluate the association of the SHR and clinical outcomes in patients with AP. We hypothesized that the SHR was an indicator to assess unfavorable outcomes of AP.

## 2. Methods

### 2.1. Study Population

A cohort of patients with AP admitted to the Pancreatitis Center of West China Hospital of Sichuan University between 1 July 2019 and 30 November 2021 was included for this study. Data were extracted from a prospectively collected database of patients with AP. This study complied with the Declaration of Helsinki and was approved by the Biomedical Ethics Review Committee of West China Hospital, Sichuan University (approval No. 2021 [320]). Informed consent was obtained from all patients.

#### Inclusion and Exclusion Criteria

Patients aged between 18 and 75 years and admitted within 72 h of symptom onset were eligible. Patients met the criteria of the diagnosis of AP if two of the following three criteria were met: (1) Abdominal pain consistent with AP (sudden onset, severe, persistent epigastric pain often radiating to the back); (2) serum lipase or amylase levels ≥3 times the upper limit of normal; (3) characteristic imaging findings (pancreatic edema, necrosis, peripancreatic fluid collections, or fat stranding) on contrast-enhanced computed tomography, magnetic resonance image, or transabdominal ultrasound [4]. The exclusion criteria included (1) pregnancy or lactation, (2) advanced or terminal-stage disease, (3) the presence of any malignancy, and (4) patients without HbA1c measurement.

### 2.2. Clinical Data Collection

The data collection followed standard operating procedures (SOPs) and was performed by experienced medical students or attending doctors, subsequently quality-checked by more senior doctors [29,30]. Demographic and clinical data were collected, including age; sex; body mass index (BMI); etiology; time to admission; Charlson Comorbidity Index (CCI); American Analgesic Association classification (ASA); referral; disease history of AP; comorbidities; key laboratory variables; and severity scores upon admission, at 24 h, 48 h, and 72 h, 5 days, and 7 days. The Modified Computerized Tomography Severity Index (MCTSI) was independently assessed by two radiologists. OF was diagnosed if the scores of each organ (respiratory, circulatory, or renal) were no less than 2 points according to the Modified Marshall Score (MMS) ≥ 2 [31]. POF was defined as a duration of organ failure lasting over 48 h [4]. Multiple organ failure was defined as no less than 2 organs manifesting functional failure. Severity classification was compiled with the revised Atlanta classification. Acute peripancreatic fluid collection (APFC) was defined as an early-phase (within 4 weeks) fluid collection adjacent to the pancreas without a well-defined wall, containing pancreatic juice, edema, and inflammatory exudate on computerized tomography imaging. Acute necrotic collection (ANC) was defined as “pancreatic necrosis or peripancreatic necrosis” on enhanced computerized tomography imaging.

### 2.3. Data Definitions

The SHR [13] was typically calculated using the following formula [32]:

(1) $$SHR=\frac{Admission\;Glucose}{(1.59\times HbA1c)-2.59}(for\;mmol/L)$$

HbA1c was measured within 3 days of admission by the Department of Laboratory Medicine, West China Hospital, using high-performance liquid chromatography (HPLC). Diabetes, prediabetes, and normal glucose status were defined according to the American Diabetes Association 2025 criteria [33]. Diabetes was diagnosed when patients met any of the following criteria: fasting blood glucose (FPG) ≥7.0 mmol/L, 2 h post-load glucose ≥11.1 mmol/L during an oral glucose tolerance test (OGTT), or HbA1c ≥ 48 mmol/mol. Prediabetes was defined as FPG levels between 5.6 and 6.9 mmol/L, 2 h glucose between 7.8 and 11.0 mmol/L on OGTT, or HbA1c values between 5.7% and 6.4%. Normal blood glucose was indicated by FPG < 5.6 mmol/L, a 2 h glucose <7.8 mmol/L on OGTT, or HbA1c < 5.7%.

### 2.4. Treatment Regimens

All patients enrolled in this study received standardized comprehensive treatment, including fluid resuscitation, nutritional support, pain management, and other symptomatic supportive therapies according to the practice guidelines of the American Gastroenterological Association (AGA) and China. Fingertip blood glucose levels were monitored, along with continuous glucose monitoring if available. AP patients with hyperglycemia were given Nihil per os initially and infused with short-acting insulin at a rate of 0.05–0.1 U/kg/h when blood glucose levels ≥16.7 mmol/L, regardless of prior DM status, and titrated to blood glucose 7.8–10 mmol/L with intravenous insulin in glucose solution. When oral intake resumed, patients were transitioned to a low-fat, diabetic-friendly diet and given subcutaneous insulin or oral agents.

### 2.5. Outcomes

The primary outcome was POF in at least one of the respiratory, cardiovascular, or renal systems. Secondary outcomes included APFC and high-dependency unit/intensive care unit (HDU/ICU) admission.

### 2.6. Statistical Analysis

Continuous variables were expressed as means ± standard deviation (SD) or medians (25–75%) based on the normality of the distribution, which was assessed using the Kolmogorov–Smirnov test. A Student’s *t*-test (normal distribution) or Mann–Whitney U-test (non-normal distribution) was used to compare the continuous variables between POF and non-POF groups. Categorical variables were presented as frequencies (percentages) and were compared using Pearson’s chi-square test or Fisher’s exact test.

Patients were stratified into four groups based on the linear chi-square test: SHR1 (≤1.03), SHR2 (1.04–1.25), SHR3 (1.26–1.46), and SHR4 (≥1.47). Restricted cubic spline (RCS) analysis with four knots at the 5th, 35th, 65th, and 95th percentiles was used to assess nonlinear associations. A threshold was identified from the spline curve, and the SHR was dichotomized accordingly. Univariable and multivariable logistic regression models were used to calculate odds ratios (ORs). In the multivariable analysis, we adjusted for the following clinically important confounders, including age, sex, BMI, time to admission, triglyceride, hematocrit, and Acute Physiology and Chronic Health Evaluation (APACHE) II on admission.

Statistical analyses were conducted using R Studio software (version 4.3.3; R Studio, Boston, MA, USA) or SPSS software (version 23; IBM SPSS Statistics, IBM, Armonk, New York, NY, USA). A two-tailed *p*-value of less than 0.05 was considered statistically significant. OR and the 95% confidence intervals (CI) were used to report the results of the univariable regression analysis. A forest plot was drawn to show the results of the multivariable logistic regression analysis.

## 3. Results

### 3.1. Baseline Characteristics

**Figure 1:**
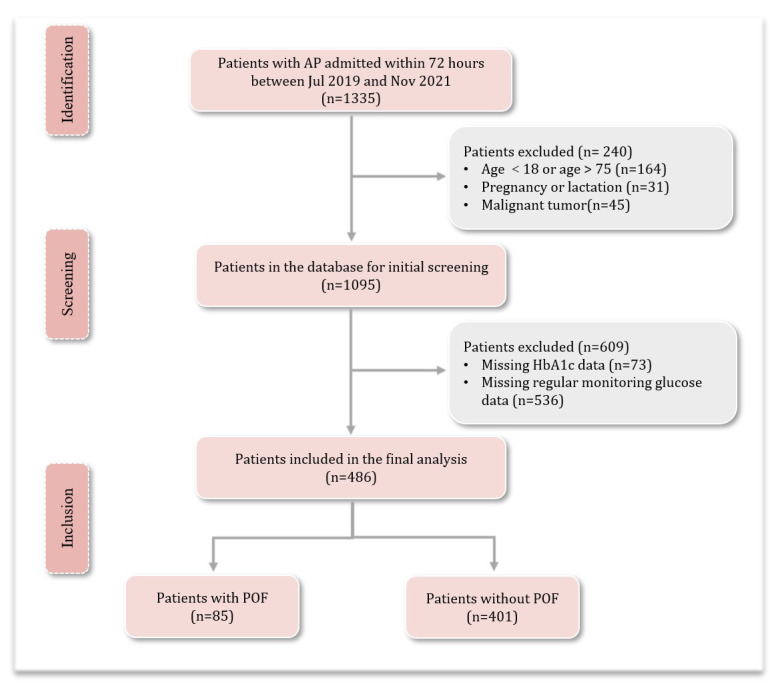
Flow chart of the study. AP, acute pancreatitis; POF, persistent organ failure.

During the study period, 1335 patients with AP were extracted from the database. Of these, 1095 patients met the inclusion criteria and were initially screened. A total of 609 patients were excluded: 536 due to missing data on regular monitoring of blood glucose and 73 due to missing HbA1c data. Finally, 486 patients with AP were included for the analysis. The comparison of the baseline and main outcomes of the data of patients with (n = 559) and without missing HbA1c data (n = 486) showed no significant difference (Supplementary Table S1). The flowchart of the study is shown in Figure 1.

Based on whether the patients had POF or not, we divided the patients into the POF (n = 401) and non-POF (n = 85) groups. The baseline clinical characteristics of the patients were compared, as shown in Table 1. The age and etiologies were insignificant between the two groups. The proportion of male patients was significantly higher in the POF group (80.0% vs. 64.3%, *p* = 0.005). Patients with POF had higher BMI, longer onset–admission intervals, and an elevated heart rate (*p* < 0.05). The difference in laboratory markers and clinical severity scores between POF and non-POF groups was significant (*p* < 0.05).

### 3.2. Association of SHR and Clinical Outcomes

**Figure 2:**
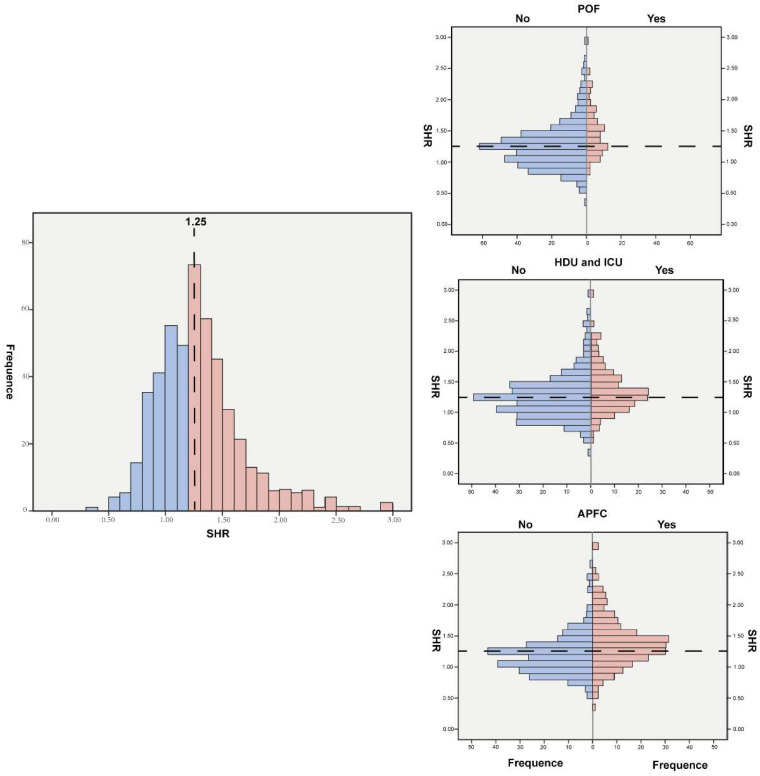
The relationship between SHR and in-hospital outcomes. SHR, stress hyperglycemia ratio.

The distribution of the SHR is shown in Figure 2. The severity of AP was stratified by SHR quartiles (Table 2). A significant correlation was observed between SHR levels and severity (*p* < 0.001), with the highest quartile showing the greatest proportion of SAP.

The clinical outcomes for the included patients were stratified by SHR levels (Table 3). Patients with higher SHR levels showed a significantly increased risk of adverse clinical outcomes, including POF ([SHR1: 7.4%, SHR2: 13.1%, SHR3: 16.4%, SHR4: 33.1%, *p* trend < 0.001]), APFC ([SHR1: 29.8%, SHR2: 37.7%, SHR3: 54.9%, SHR4: 67.8%, *p* trend < 0.001]), and the need for HDU/ICU admission ([SHR1: 21.5%, SHR2: 29.5%, SHR3: 37.7%, SHR4: 42.1%, *p* trend < 0.001]). Additionally, the length of stay (LOS) was significantly increased, with a higher SHR (*p* trend < 0.001). No significant association was found between the SHR and the occurrence of ANC or pancreatic infection (*p* > 0.05).

**Figure 3:**
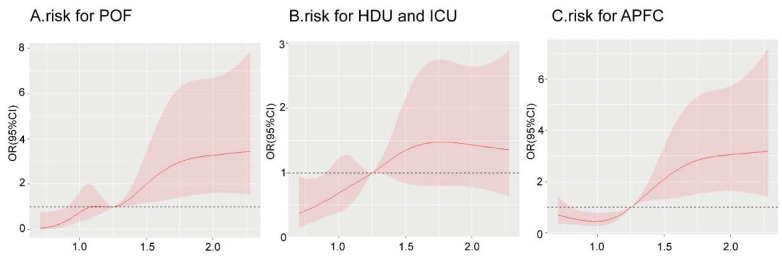
Distribution of SHR levels (Overall, POF, HDU and ICU, and APFC). HDU/ICU, high dependency unit/intensive care unit; APFC, acute peripancreatic fluid collection.

To better illustrate the association between SHR and clinical outcomes, we modeled the SHR using RCS to provide a dose–response relationship. As shown in Figure 3, the SHR was associated with the risk of POF (Figure 3A, *p*-value for nonlinear spline terms = 0.148) and HDU/ICU admission (Figure 3B, *p*-value for nonlinear spline terms = 0.256). Notably, a significant curvilinear association was observed between the SHR and the risk of APFC (Figure 3C, *p*-value for nonlinear spline terms = 0.009), suggesting a nonlinear dose–response relationship.

A threshold value of SHR = 1.25 was identified through the combined analysis of RCS and quartile stratification. Based on this threshold, we divided patients into the low-SHR group (SHR ≤ 1.25) and the high-SHR group (SHR > 1.25).

### 3.3. Logistic Analysis

**Figure 4:**
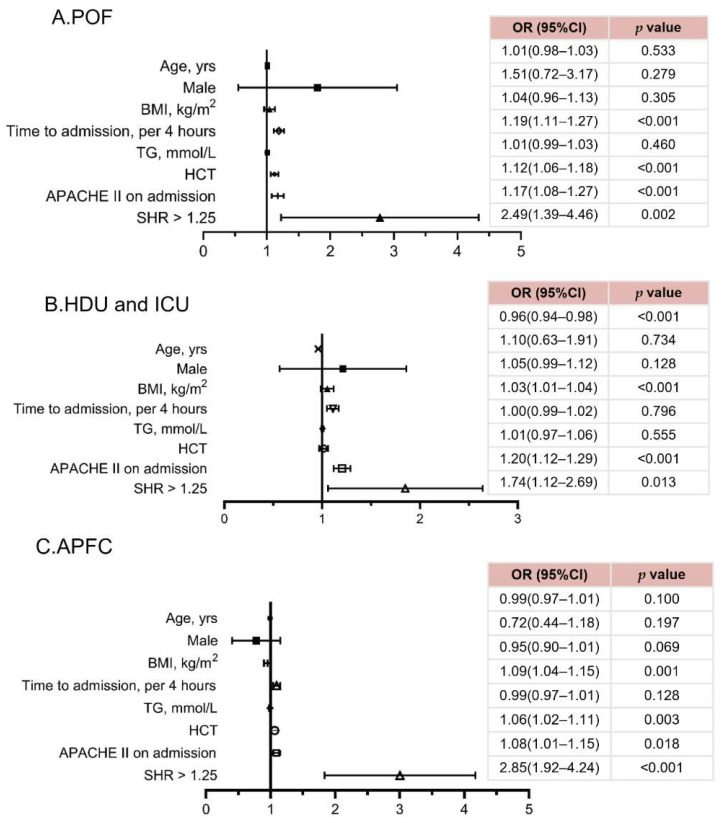
Forest plots showing correlated factors for in-hospital clinical outcomes: (**A**) POF, (**B**) HDU and ICU, and (**C**) APFC, as determined by multivariable logistic regression analysis. Note: POF, persistent organ failure; HDU/ICU, high dependency unit/intensive care unit; APFC, acute peripancreatic fluid collection; APACHE, Acute Physiology and Chronic Health Evaluation; SHR, stress hyperglycemia ratio.

Univariable logistic regression analysis was performed to identify the risk factors for the outcome in this cohort of patients. The results indicated that factors such as age, gender, BMI, onset–admission intervals, hematocrit, triglyceride, APACHE II, and SHR > 1.25 were significantly associated with the occurrence of POF, ICU/HDU admission, and APFC (Table 4). The potential confounding factor and multicollinearity between the SHR and APACHE II, which includes serum glucose in its calculations, was analyzed. The results of the analysis showed variance inflation factor was between one and two, indicating that there was no multicollinearity problem. No significant association was found between the SHR and the occurrence of ANC or pancreatic infection (*p* > 0.05). After adjusting for potential confounders in multivariable analysis, a high SHR remained independently associated with POF (OR: 2.49, 95% CI: 1.39–4.46, *p* = 0.002), ICU/HDU admission (OR: 1.74, 95% CI: 1.12–2.69, *p* = 0.013), and APFC (OR: 2.85, 95% CI: 1.92–4.24, *p* < 0.001) (Figure 4). Onset–admission intervals (*p* < 0.001) and APACHE II score (*p* < 0.05) were significantly associated with the occurrence of POF, ICU/HDU admission, and APFC and were adjusted for in the multivariable analysis.

## 4. Discussion

In this study, we investigated the role of a new parameter, the SHR, for the prognosis of AP in a cohort of 486 patients. The results for the first time identified a high SHR as an independent predictor of adverse outcomes in AP, including POF, APFC, and HDU/ICU admission.

Patients with AP commonly suffer from hyperglycemia, even if they have no pre-existing diabetes. The causes of hyperglycemia in AP include pancreatic islet cell damage due to inflammation and necrosis of the pancreas, insulin resistance induced by pro-inflammatory cytokines and stress hormones, excessive counter-regulatory hormones, exocrine–endocrine crosstalk, etc. Of these, stress plays a significant role in the induction of hyperglycemia. Stress in AP activates both the hypothalamic–pituitary–adrenal axis and the sympathetic–adrenal medullary system; leads to the release of multiple counter-regulatory hormones such as epinephrine, norepinephrine, cortisol, and glucagon; promotes hepatic glycogenolysis and gluconeogenesis; and thereby elevates blood glucose levels. A proinflammatory cytokine cascade, including interleukin-6, tumor necrosis factor-alpha, and Interleukin-1 beta, disrupts insulin signaling pathways and leads to insulin resistance. Together, these mechanisms result in the development of SH.

SH is considered an adaptive immune–neurohormonal response to stress, providing metabolic substrates to struggling organs. However, it is also responsible for a series of detrimental effects. Nowadays, studies have demonstrated that SH is associated with increased morbidity and adverse outcomes for critically ill patients. Current research [8] has primarily focused on blood glucose levels on admission for AP, but this single-time-point measurement does not effectively differentiate between true SH and underlying chronic dysglycemia. Furthermore, our study [11] identified that SH persisting over 48 h was associated with the prognosis of AP. Nevertheless, SH is a transient disturbance in glucose metabolism that fluctuates rapidly. This dynamic nature highlights the importance of timely glucose monitoring and appropriate management strategies.

Few studies have been conducted on patients with AP associated with SH, and there is a lack of a standardized definition for SH in AP. Previous research has primarily focused on blood glucose levels at a single time point and the pre-existence of diabetes. As we know, blood glucose measurement provides a simple and quick assessment, and HbA1c offers valuable insights into long-term glucose control. However, the independent utilization of these indicators has significant limitations. The ideal indicator of SH needs to accurately capture the acute stress-induced blood glucose response and distinguish SH from chronic hyperglycemia. The levels of blood glucose in AP need to be interpreted in the context of the disease and previous glycemic status. The SHR [13], which combines acute-phase blood glucose with chronic glycemic status measured by HbA1c, offers a real-time, quantitative evaluation of glycemic imbalance and a more comprehensive assessment of the glycemic response to stress. A higher SHR indicates greater SH, suggesting a stronger acute metabolic derangement rather than chronic hyperglycemia or diabetes mellitus. The SHR has already shown strong prognostic value in cardiovascular diseases [34,35], kidney injury [17], ischemic stroke [25], sepsis [25], etc.

The SHR has demonstrated robust prognostic value in critical illnesses. Our study demonstrates a significant correlation between SHR levels and the outcomes of AP. As the levels of the SHR increased, the incidence of POF, APFC, and HDU/ICU admission increased correspondingly. These findings suggest that the SHR may serve as a valuable prognostic biomarker for the severity of AP. RCS analysis revealed a significant nonlinear relationship between the SHR and APFC (*p* = 0.009). Specifically, when the SHR was below 1.25, the OR of APFC was less than 1. This indicated that mild SH at lower SHR levels may have a compensatory effect, which helps to preserve pancreatic function and reduce acute fluid collection. We hypothesize that short-term SH may offer protective benefits, as the body may utilize mild hyperglycemia to counterbalance the inflammatory response. When the SHR exceeded 1.25, the OR value increased above 1, indicating that higher SHR levels are strongly associated with an increased risk of APFC. This suggests that significant hyperglycemia may exacerbate pancreatic injury as a pathological factor. These findings highlight SHR = 1.25 as a critical threshold, providing insights into the dual role of hyperglycemia in AP.

To date, POF has a very high mortality in AP, and its prediction in the early stage is crucial. However, early identification of high-risk patients for POF is still challenging. Studies and systematic reviews [29,36,37,38] indicated that albumin, high-density lipoprotein–cholesterol, Interleukin-6, GDF15, a deep learning model, the Ranson Score, and the bedside index for severity in AP are effective predictors for POF in patients with AP. Here, the SHR has emerged as a new valuable early marker, integrating both early glycemic control and admission glucose levels to guide the development of individualized glucose management strategies. Interestingly, an SHR less than 1.25 showed a trend toward a lower risk for POF, although these results were not statistically significant. It is suggested that hyperglycemia at lower SHR levels may not substantially affect the risks of POF and could be influenced by other clinical factors. An SHR > 1.25 was significantly associated with POF in multivariable analysis. Even after adjusting for potential confounders, a high SHR remained an independent risk factor, suggesting that the SHR is a reliable predictor of adverse clinical outcomes in patients with AP. Pre-existence or newly diagnosed diabetes was not significantly associated with POF, which was consistent with the results of our previous study [7]. This suggests that the SHR plays a more direct role in predicting adverse clinical outcomes in AP, independent of diabetes status.

Based on our previous research [11], which demonstrated the association between the duration of SH and mortality, the current study focuses on the intensity of SH, specifically as reflected by SHR levels, and its relationship with clinical outcomes in AP. We established a more precise clinical threshold of SHR = 1.25, providing stronger evidence of the link between the SHR and AP outcomes. While the duration of hyperglycemia requires long-term monitoring, the SHR serves as an immediate warning signal, facilitating early intervention to guide treatment decisions. This integrated approach—using SHR levels for timely action and the duration of hyperglycemia for sustained management—could offer more accurate guidance for blood glucose control in AP patients, ultimately helping to prevent poor prognoses.

There are several clinical implications of this study. As hyperglycemia is closely associated with patient prognosis, timely glucose-lowering therapy has reached consensus. Prompt recognition of SH in high-risk patients can help improve inpatient management. The SHR helps to identify patients at higher risk of complications of AP, and patients with an SHR over 1.25 may benefit from tighter glycemic control. Regarding treatment guidance, using the SHR offers the advantage of providing more personalized treatment plans. It enables the precise identification of patients who require glucose intervention at the time of admission, allowing for the establishment of a comprehensive glucose management plan that includes blood glucose monitoring, nutritional support, fluid resuscitation, and glucose-lowering medication. This approach helps reduce overtreatment and ensures timely intervention. Unlike random hyperglycemia, the SHR adjusts for baseline glycemic status (HbA1c), making it a more precise prognostic tool for AP. Patients with SH who have recovered from the acute illness should be followed up for the risk of diabetes. For those acutely ill patients with haemodilution, altered erythropoiesis, red blood cell turnover, and glycaemic variability affecting HbA1c-glucose relationships, the applicability of the SHR should be interpreted with caution and handled accordingly.

There are several limitations to this study. Firstly, although we identify the role of the SHR in the prognosis of AP, the observational study cannot explain the cause-and-effect relationship. The underlying mechanisms of SH-induced organ dysfunction have not been thoroughly investigated. Possible explanations underly increased glucose levels that enhance reactive oxygen species production, exacerbate oxidative stress and microvascular injury, and lead to endothelial dysfunction and organ failure. However, this hypothesis requires further exploratory research. Secondly, this is a single-center study, requiring further studies to generalize our findings. Lastly, the follow-up period data were limited, only covering outcomes during hospitalization and three months after discharge. Further studies are needed to assess the long-term survival and quality of life.

## 5. Conclusions

In summary, our study demonstrates that an SHR greater than 1.25 is independently associated with adverse outcomes in AP, including POF, APFC, and HDU/ICU. As a simple and readily available biomarker that integrates both acute glucose elevation and chronic glycemic status, the SHR may help to improve early risk stratification and guide more individualized glucose management strategies in patients with AP.

## Figures and Tables

**Table 1:** Baseline clinical characteristics of the patients.

Variables	Non-POF (n = 401)	POF (n = 85)	*p* Value
Age, years	45 (37.5–53.5)	47 (38.5–52.5)	0.995
Male, n (%)	258 (64.3)	68 (80.0)	0.005
BMI, kg/m^2^	25.73 (23.51–28.34)	26.90 (24.80–29.05)	0.006
Time to admission, hours	20 (12–39)	32 (24–49)	<0.001
Etiology, n (%)			0.505
Biliary	95 (23.7)	16 (18.8)	
Hyperlipidemia	174 (43.4)	35 (41.2)	
Alcohol	15 (3.7)	5 (5.9)	
Idiopathic	79 (19.7)	16 (18.8)	
Mixed	28 (7.0)	11 (12.9)	
Other	10 (2.5)	2 (2.4)	
Laboratory markers *			
Hematocrit, L/L	0.43 (0.39–0.46)	0.46 (0.42–0.51)	<0.001
White blood cell counts, ×10^9^/L	12.91 (10.00–15.70)	12.34 (10.46–16.98)	0.596
Albumin, g/L	42.2 (38.0–45.5)	35.4 (31.3–39.4)	<0.001
Serum glucose, mmol/L	9.9 (7.4–13.5)	11.0 (8.7–14.1)	0.028
Blood urinary nitrogen, mmol/L	4.8 (3.8–5.9)	4.8 (3.7–6.6)	0.258
Creatinine, mmol/L	64 (52–80)	76 (62–100)	<0.001
Triglycerides, mmol/L	6.98 (1.64–15.08)	6.04 (1.48–15.03)	0.724
lactate dehydrogenase, U/L	241 (186–352)	467 (325–644)	<0.001
Calcium ions, mmol/L	2.18 (2.07–2.27)	1.92 (1.61–2.06)	<0.001
C-reactive protein, g/L	108.5 (35.6–211.8)	309 (177.5–402.3)	<0.001
Interleukin 6	67.9 (28.0–151.5)	217 (91.5–381.5)	<0.001
Procalcitonin	0.19 (0.09–0.55)	1.52 (0.48–4.01)	<0.001
Amylase	268 (99–723)	474 (265–857)	<0.001
Lipase	522 (157–1383)	774 (399–1532)	0.009
*p*-amylase	214 (64.5–604)	414 (219–805)	<0.001
Clinical severity scores *			
Glasgow	1 (0–2)	2 (1.5–3)	<0.001
SIRS	2 (1–2)	2 (2–3)	<0.001
BISAP	1 (1–2)	2 (1–3)	<0.001
MMS	1 (0–2)	3 (1–4)	<0.001
APACHE II	5 (3–7)	7 (5–10)	<0.001

* Values are median (IQR). Note. POF, persistent organ failure; P-amylase, pancreas amylase; BISAP, bedside index for severity in acute pancreatitis; SIRS, systemic inflammatory response syndrome; MMS, modified Marshall score; APACHE II, Acute Physiology and Chronic Health Evaluation II.

**Table 2:** Comparison of the severity among the patients divided into four quartiles based on the stress hyperglycemia ratio.

Variable	Total (n = 486)	Stress Hyperglycemia Ratio					*p* Trend
		≤1.03 (n = 121)	1.04–1.25 (n = 122)	1.26–1.46 (n = 122)	≥1.47 (n = 121)	*p* Value	
Severity						<0.001	<0.001
Mild	191 (39.3)	70 (57.9)	51 (41.8)	43 (35.2)	27 (22.3)		
Moderately severe	210 (43.2)	42 (34.7)	55 (45.1)	59 (48.4)	54 (44.6)		
Severe	85 (17.5)	9 (7.4)	16 (13.1)	20 (16.4)	40 (33.1)		

**Table 3:** Comparison of clinical outcomes among the patients divided into four quartiles based on the stress hyperglycemia ratio.

Variable	Total (n = 486)	Stress Hyperglycemia Ratio					*p* Trend
		≤1.03 (n = 121)	1.04–1.25 (n = 122)	1.26–1.46 (n = 122)	≥1.47 (n = 121)	*p* Value	
Organ failure						<0.001	<0.001
None	312 (64.2)	99 (81.8)	75 (61.5)	79 (64.8)	59 (48.8)		
Transient	89 (18.3)	13 (10.7)	31 (25.4)	23 (18.9)	22 (18.2)		
Persistent	85 (17.5)	9 (7.4)	16 (13.1)	20 (16.4)	40 (33.1)		
APFC	231 (47.5)	36 (29.8)	46 (37.7)	67 (54.9)	82 (67.8)	<0.001	<0.001
ANC	84 (17.3)	19 (15.7)	20 (16.4)	18 (14.8)	27 (22.3)	0.397	0.260
IPN	13 (2.7)	1 (0.8)	4 (3.3)	3 (2.5)	5 (4.1)	0.362	0.210
HDU/ICU	159 (32.7)	26 (21.5)	36 (29.5)	46 (37.7)	51 (42.1)	0.003	<0.001
Surgery	27 (5.6)	4 (3.3)	8 (6.6)	5 (4.1)	10 (8.3)	0.302	0.185
LOS	10 (7–13)	8 (6–11)	8.5 (6–12)	10 (7–14)	13 (9–17)	<0.001	<0.001

Note: APFC, acute peripancreatic fluid collection; ANC, acute necrotic collection; IPN, infected pancreatic necrosis; HDU/ICU, high dependency unit/intensive care unit; LOS, length of hospital stay.

**Table 4:** Univariate logistic regression for in-hospital outcomes.

Variables	POF		HDU and ICU		APFC	
	OR (95%CI)	*p* Value	OR (95%CI)	*p* Value	OR (95%CI)	*p* Value
Age, years	0.99 (0.98–1.01)	0.560	0.97 (0.95–0.98)	<0.001	0.99 (0.98–1.01)	0.348
Male, n (%)	2.22 (1.26–3.92)	0.006	1.26 (0.84–1.90)	0.272	0.86 (0.59–1.26)	0.445
Body mass index, kg/m^2^	1.09 (1.03–1.17)	0.006	1.09 (1.03–1.15)	0.002	0.97 (0.93–1.02)	0.289
Time to admission, hours	1.03 (1.02–1.05)	<0.001	1.03 (1.01–1.04)	<0.001	1.02 (1.01–1.03)	<0.001
Hematocrit	1.14 (1.09–1.20)	<0.001	1.04 (1.01–1.08)	0.013	1.04 (1.01–1.08)	0.006
hypertriglyceridemia	0.99 (0.98–1.02)	0.691	1.01 (0.99–1.02)	0.366	0.98 (0.97–0.99)	0.026
Pre-existing diabetes	0.68 (0.42–1.10)	0.119	0.69 (0.47–1.01)	0.055	0.78 (0.55–1.11)	0.177
APACHE II score	1.21 (1.12–1.29)	<0.001	1.16 (1.09–1.22)	<0.001	1.09 (1.03–1.15)	0.002
SHR > 1.25	2.80 (1.69–4.65)	<0.001	1.89 (1.29–2.78)	0.001	3.01 (2.08–4.36)	<0.001

Note: POF, persistent organ failure; HDU/ICU, high dependency unit/intensive care unit; APFC, acute peripancreatic fluid collection; APACHE, Acute Physiology and Chronic Health Evaluation; SHR, stress hyperglycemia ratio.

## Data Availability

The original contributions presented in this study are included in the article/Supplementary Material. Further inquiries can be directed to the corresponding author(s).

## Supplementary Materials

The following supporting information can be downloaded at: https://www.mdpi.com/article/10.3390/jcm14144970/s1, Table S1. Baseline characteristics between the patients with and without missing HbA1c data.
