# Supplementary material for: The Stress Hyperglycemia Ratio as a Predictor of Clinical Outcomes in Acute Pancreatitis: A Retrospective Cohort Study
Source: J Clin Med. 2025 Jul 14;14(14):4970. doi: 10.3390/jcm14144970 (PMC12294952; doi:10.3390/jcm14144970)
Supplement: Supplementary file 1 [file jcm-14-04970-s001.zip › jcm-3704340-supplementary.pdf]

Supplementary Table S1. Baseline characteristics between the patients with and without missing HbA1c data

**Variables**	**Patients excluded missing HbA1c (n = 486)**	**Patients included missing HbA1c (n = 559)**	***P* value**
Age, years	46 (38-53)	46 (37.5-53)	0. 875
Male, n (%)	312 (64.2)	370 (66.2)	0.593
BMI, kg/m^2	25.91 (23.63-28.40)	26.99 (22.44-28.18)	0.452
Laboratory			
HCT	0.43 (0.40-0.47)	0.43 (0.40-0.46)	0.556
WBC	13.13 (10.13-15.78)	13.36 (11.22-16.78)	0.418
ALB	40.44 (36.60-45.03)	40.40 (37.50-44.60)	0.903
GLU	10.91 (7.69-13.55)	10.58 (6.46-9.77)	0.227
BUN	4.8 (3.8-6.0)	4.5 (3.6-6.3)	0.940
Creatine	66 (54-82)	68 (51-85)	0.594
LDH	263 (190-416)	234 (188-373)	0.158
CRP	155.75 (45.7-261.8)	160.99 (23.9-121.5)	0.555
IL6	164.67 (35.6-198.0)	164.79 (26.0-145.5)	0.995
Severity			0.586
mild	191 (39.3)	222 (39.7%)	
moderately	210 (43.2%)	242 (43.2%)	
severe	85 (17.5%)	95 (17. 1%)	
APFC	74 (15.2%)	95 (16.9%)	0.520
IPN	14 (2.5%)	15 (2.7%)	0.735
